# Comparative Study of Contact Repulsion in Control and Mutant Macrophages Using a Novel Interaction Detection †

**DOI:** 10.3390/jimaging6050036

**Published:** 2020-05-20

**Authors:** José Alonso Solís-Lemus, Besaiz J Sánchez-Sánchez, Stefania Marcotti, Mubarik Burki, Brian Stramer, Constantino Carlos Reyes-Aldasoro

**Affiliations:** 1 School of Biomedical Engineering and Imaging Sciences, King’s College London, London SE1 7EH, UK; 2 Randall Centre for Cell & Molecular Biophysics, King’s College London, London SE1 1UL, UK; besaiz.sanchez_sanchez@kcl.ac.uk (B.J.S.-S.); stefania.marcotti@kcl.ac.uk (S.M.); zile.burki@kcl.ac.uk (M.B.); brian.m.stramer@kcl.ac.uk (B.S.); 3 GiCentre, Departmen t of Computer Science, School of Mathematics, Computer Science and Engineering, City, University of London, London EC1V 0HB, UK

**Keywords:** cell segmentation, cell tracking, macrophages, cell shape, contact analysis

## Abstract

In this paper, a novel method for interaction detection is presented to compare the contact dynamics of macrophages in the *Drosophila* embryo. The study is carried out by a framework called macrosight, which analyses the movement and interaction of migrating macrophages. The framework incorporates a segmentation and tracking algorithm into analysing the motion characteristics of cells after contact. In this particular study, the interactions between cells is characterised in the case of control embryos and *Shot* mutants, a candidate protein that is hypothesised to regulate contact dynamics between migrating cells. Statistical significance between control and mutant cells was found when comparing the direction of motion after contact in specific conditions. Such discoveries provide insights for future developments in combining biological experiments with computational analysis.

## 1. Introduction

Cellular migration is essential in many biological phenomena, both during development and in adult life. This process plays a key role in both physiological (such as embryogenesis [1,2], angiogenesis [3], and inflammation [4,5]) and pathological conditions (such as wound healing [6,7] and cancer invasion [8,9,10]). This work focuses on a specific migratory cell type of the immune system, macrophages. These cells have multiple roles, such as maintenance of homoeostasis [11], tissue repair [12], and immune response to pathogens [13]. Misregulation of macrophages migratory patterns can be related to autoimmune disease and cancer.

*Drosophila melanogaster*, also known as the common fruit fly, has been widely studied as a model organism [14,15,16]. Although in evolutionary terms, the fly is very far from vertebrates, it shares many developmental and cellular processes with other organisms, including humans [17]. Thus, investigations with *Drosophila* have led to insights about the role of macrophages and how they integrate migratory movement with external cues [18].

A specific process called contact inhibition of locomotion has been described [19], which involves specific cytoskeletal interactions between cells to allow functional migration of macrophages. In particular, microtubules have been shown essential for this process [19,20].

Although the relevance of the actin-microtubule network during cell motility has been previously reported, less is known about the role of the network regulators [21]. *Shot* (shortstop or spectraplakin short stop) works as an actin-microtubule crosslinker [22,23] and regulates microtubule polarisation [24]. This process is necessary to maintain the microtubule dynamics in the macrophage lamellae, which also leads to an alteration in macrophage polarity and migration [19]. In the present study, macrophages from control embryos were compared to *Shot3* mutant embryos (referred to as mutants), to evaluate differences in their migration dynamics. We focus our study on the analysis of the contact inhibition of locomotion, because this is a very well-established tool to analyse the capacity of cells to react after contacting.

Tracking of cells comprises the identification of the cells from background and then linking between previously detected cells in one time frame to the same cells in subsequent frames. In this work, tracking will be defined as a function of segmentation, that is the correct identification of each cell from the background and, probably more important, and from the other cells. Both segmentation and tracking of cells have been widely studied with many imaging modalities [25,26,27,28]. Cell tracking when cells are observed with phase contrast microscopy was presented in [25,26], showing quantitative analysis of cell dynamics in vitro. In [27,28], several tracking methodologies were evaluated with a number of migratory cells under different conditions. The methodologies were compared, not only in their ability to track the cells that were segmented, but also to identify events like mitosis. Other cellular events, e.g., interactions between cells, are also of huge importance as these may be related to communication between cells or cell signalling. To study these events, a more thorough study of a tracks’ features is necessary.

Movement analysis in this work will be defined as the analysis of features derived from tracks and will be performed to examine specific research questions related to certain phenomena to be studied. For instance, in [29], tracks were classified depending on certain features, e.g., curvature and speed. In a related work, a movement pattern analysis provided insights about a toxicological environment assessment with *Artemia Franciscana* swimming in chambers with sub-lethal doses of potassium dichromate [30]. In that experiment, the tracks produced by the movement of these marine crustaceans were examined for specific patterns of migration (circular motions), which were related to the levels of toxicity. Contributions regarding the specific data analysed in this work have been varied. Segmentation of macrophages into single frames was presented in [31], showcasing the complex interactions that manifest such as overlapping (*clumps*). In [32], the relationship between contiguous frames was incorporated into the segmentation of single cells, allowing for a controlled measurement of shape parameters between overlapping events. Finally, macrosight, a software framework to analyse the movement and the shape variation of fluorescently-labelled macrophages, was presented in [33], where overlapped clumps were considered moments of assumed interaction between the cells and thus the movement before and after contact was analysed.

**Figure 1:**
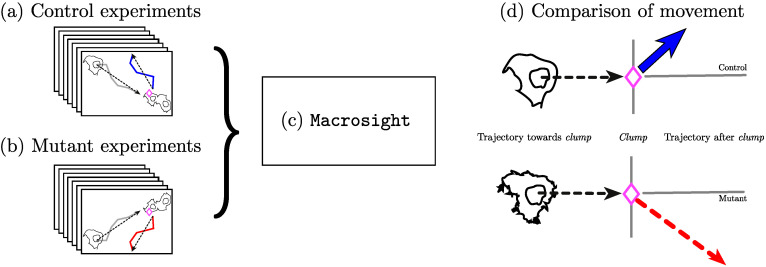
Illustration of the main hypothesis in this work. Different movement patterns from control and mutant samples are expected as a result of the movement analysis performed. The diagram shows the two different types of cells: controls (**a**) and mutants (**b**) being processed with macrosight [33] (**c**). The output (**d**) consists of measurements of the cell’s trajectories and the changes in direction upon interactions represented by the different types of line and colours in the diagram.

The macrosight software is used to search for an underlying difference in the movement between control and mutant cells. The main contribution consists of the use of a software framework to provide robust, quantitative measurements of the same object in different conditions. It is worth noting that the two main hypotheses of macrosight are (i) that cell-cell contact accounts for an interaction between cells and (ii) as a result of an interaction, one or both cells involved in the interaction will noticeably change direction before and after contact. Furthermore, Figure 1 shows a graphical abstract of the main contribution of the comparison between control and mutant experiments.

A preliminary version of this work was presented at the 23rd Medical Image Understanding and Analysis (MIUA) [34]. The algorithms have been extended, and several new experiments with new data are presented. Thus, this work now describes the following topics, not included previously: (i) a more thorough explanation of the interactions of macrophages and a stronger description of the methodology; (ii) a new representation of the distribution of angles, allowing a much better interpretation of the results; and (iii) a more thorough literature review of the problem.

The code corresponding to this work was developed in the programming environment of Matlab^®^ (The Mathworks^TM^, Natick, MA, USA) and is available as open source from the following repository: https://github.com/alonsoJASL/macrosight. The repository contains several test sets. In addition, one dataset is available from the repository Zenodo [35].

## 2. Materials

In this work, a total of 16 time sequences of macrophages in *Drosophila* embryos were analysed. Of these, 5 corresponded to controls, and 11 corresponded to *Shot3* mutant samples. One control and three mutants were discarded, as will be explained below, and the final number of time sequences analysed was fourteen.

### 2.1. Fly Stocks and Preparation

We visualised macrophages in the embryo by using the UAS/GAL4 system [36]. We used the srpHemo-Gal4 driver [37], which mediates the expression of genes downstream of a UAS sequence specifically in macrophages, to express the following UAS fluorescent probes: UAS-RedStinger for the nuclei [38] and UAS-Clip-GFP for the microtubules [19]. The control and *Shot3* mutant genotypes used were *w; ; srpHemo-Gal4, UAS-RedStinger, UAS-Clip-GFP* and *w; Shot3; srpHemo-Gal4, UAS-RedStinger, UAS-Clip-GFP*, respectively. To obtain the appropriate embryo stage, adult flies were left to lay eggs overnight on grape juice agar plates at room temperature. Embryos were dechorionated in bleach. Embryos of the appropriate genotype were identified based on the presence of fluorescent probes and/or the absence of balancer (control) chromosomes expressing fluorescent markers. Dechorionated embryos were mounted in 10S Voltalef oil (VWR) on a glass coverslip covered with heptane glue and a gas-permeable Lumox culture dish (Sarstedt), as described previously [16].

### 2.2. Microscopy

The macrophages were observed with fluorescence microscopy following the protocol described in [19,39]. Embryos were mounted as previously described [20], and time-lapse images of developmental dispersal (Developmental Stages 14–16) were acquired every 10 s with a PerkinElmer Ultraview spinning disk microscope, equipped with a ×63 NA 1.4 Plan-Apochromat oil objective.

The nuclei were labelled in red, and the microtubules were labelled in green. Each image of a time-lapse sequence was acquired every ten seconds, and the lateral dimensions of the pixel were 0.21 μm. The dimensions of the images of all the experiments were $\left(n_{w},n_{h},n_{d}\right)=\left(512,672,3\right)$ (rows, columns, channels).

**Figure 2:**
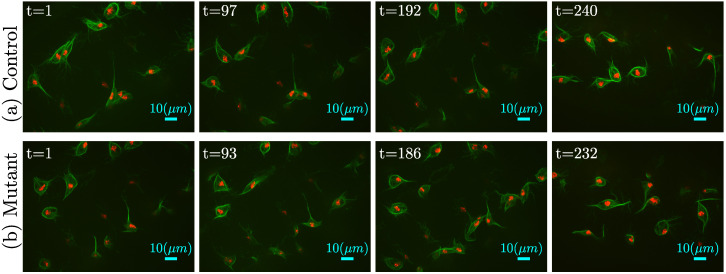
Comparison between four frames of (**a**) the control against four frames of a (**b**) mutant dataset. These datasets were selected as they had a similar number of frames, and thus, a similar spacing between the frames in both cases could be shown (≈95).

The number of time frames of the control datasets ranged between 137 and 272, whilst for the mutant, it was between 135 and 422 frames. Figure 2 shows a comparison with four frames of one control and one mutant.

## 3. Methods

In this work, two or more cells close enough so that their microtubules were in contact and overlapped were defined as a *clump*. More specifically, when the green and red fluorescent channels were segmented separately and when the green channels of two cells overlapped and were segmented as a single region of connected pixels and contained two separate segmented regions of the red channel, this was considered a *clump*.

**Figure 3:**
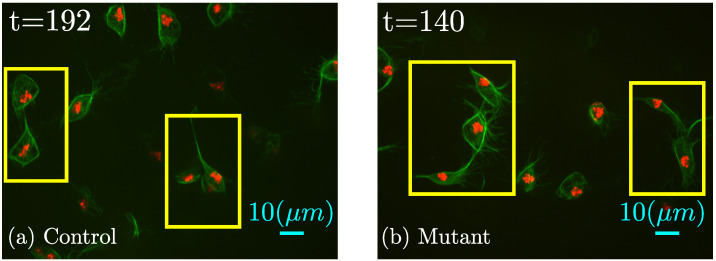
Illustration of a series of *clumps* in (**a**) control and (**b**) mutant experiments. Both datasets present overlapping events, i.e., *clumps*, which are highlighted with yellow boxes. It should be noted that although the microtubules are overlapping, the nuclei are still separated.

These *clumps* are very important for the study of interactions caused by cell-cell contact, as presented in Figure 3.

Cells have been shown to align their microtubules and change the orientation of movement drastically [19]. The contact observed in certain *clumps* suggests a change of direction of the migration patterns of those cells involved in the contact. This type of interaction was analysed previously in [33], where cell-cell contact was shown to influence the movement of cells.

**Figure 4:**
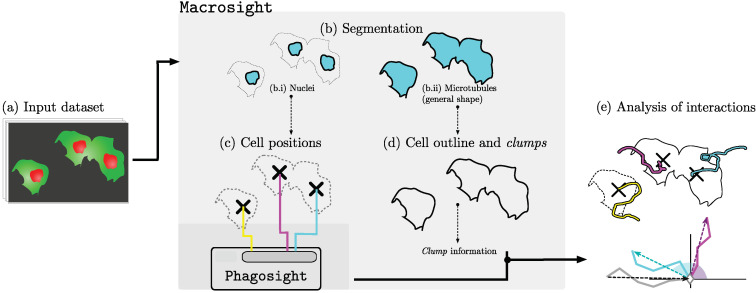
Illustration of the macrosight framework parts used in this work. (**a**) Illustration of a sequence of images with cells with red nuclei and green microtubules. The two fluorescent channels are segmented in (**b**) based on a hysteresis threshold where the levels are selected by the Otsu [41] algorithm. The segmentation of the red channel (**b.i**) provides the cell positions necessary to produce (**c**) the tracks of the cells using the keyhole tracking algorithm [40] (represented in cyan, magenta, and yellow). Finally, the tracks’ information is combined with the clump information (**d**) from the segmented green channel (**b.ii**) to allow analysis of movement based on contact events (**e**), producing the change of direction chart per cells in the clump. In this case, two cells interact and form a clump (magenta and cyan), whilst the other cell (yellow) does not form a clump. The diagram illustrates the change of direction of those cells that interact in the clump.

Macrosight [33] is a framework for the analysis of moving macrophages capable of segmenting the two channels that form the fluorescent image (e.g., red and green) in the dataset presented previously and applies the keyhole tracking algorithm inside the PhagoSight framework [40] on the centroids of the segmented nuclei. Figure 4 shows an illustration of the flow of information in macrosight. Each track generated $T_{r}$ contains information on the (i) position $x_{t}$ at a given time frame *t*, (ii) track identifier *r*, (iii) velocity $v_{t}$, and whether the cell is part of a clump.

Each *clump* can be uniquely identified through an individual code c(r,q), where r>q indicates that at a certain time frame *t*, tracks $T_{r}$ and $T_{q}$ belong in the same clump. The time frames during which the cells overlap and form a clump are denoted by $t_{k_{0}},t_{k_{1}},\cdots ,t_{k_{C}}$. The tracks $T_{r}$ and $T_{q}$ will be observed for *S* frames before $t_{k_{0}}$ and until at *S* frames after $t_{k_{C}}$. Frames $t_{k_{0}-S},\cdots ,t_{k_{C}+S}$ will be referred to as the *clump span*; likewise, the time frames where the tracks are interacting, $t_{k_{0}},t_{k_{1}},\cdots ,t_{k_{C}}$, will be referred to as *time in clump*.

**Figure 5:**
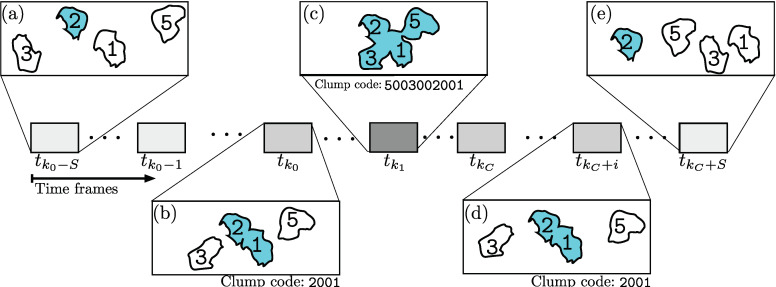
Illustration of clump codes for the different time frames for a particular track $T_{2}$. The horizontal axis represents the time, and the detail of five frames is presented to illustrate the evolution of track $T_{2}$ as it interacts with other cells. In (**a**,**e**), track $T_{2}$ is not in contact with any other cell, thus no clump is present. (**b**,**d**) Represent moments when $T_{2}$ and $T_{1}$ interact in clump 2001. Following, in (**c**), tracks $T_{3}$ and $T_{5}$ become present in the clump; thus, the *clump* code changes to 5003002001.

Several tracks can join together into a single clump; thus, the *clump* codes evolve. Figure 5 illustrates the evolution of a given track $T_{2}$ and its involvement in two different clumps as a cartoon.

**Figure 6:**
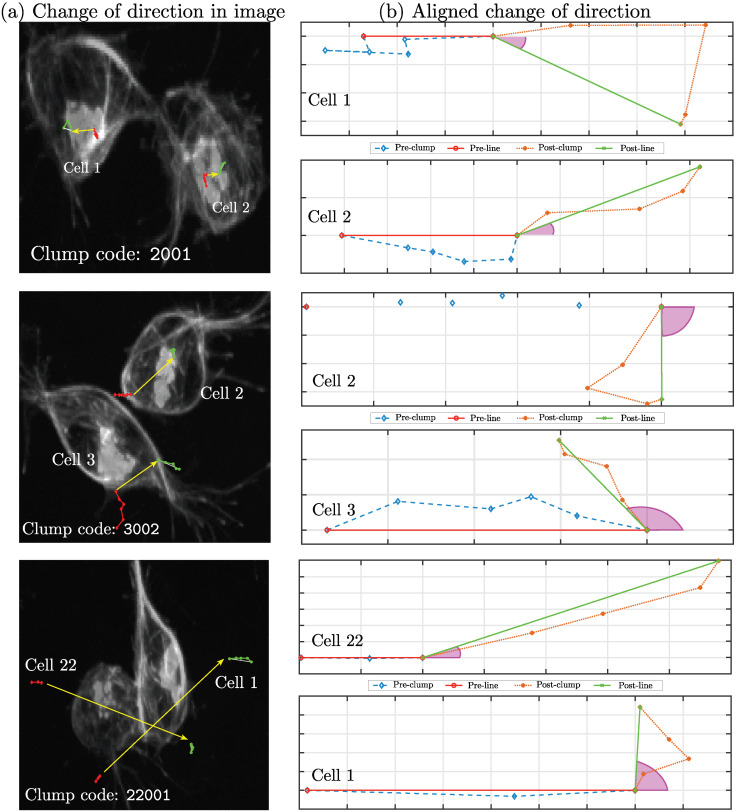
This figure shows three examples of the change of direction before and after a clump. Column (**a**) shows the cells that interact in three different clumps: 2001, 3002, and 22001. A red line (*−) shows the orientation of movement before the clump, and a green line (⋄−) represents the positions of movement after. A yellow arrow is superimposed on the image to show the trajectory of the cell inside the clump. (**b**) Simplified view of the cells’ changes in orientation. The cells’ path before the clump is represented in blue (−⋄−). The path of the cell after the clump is shown in orange (:*). The angle arc of orientation is shown in magenta. Notice that the movement of the two cells involved in clump 2001 is considerably smaller compared to the other cases.

To provide the reader with a real representation of the cell movements, Figure 6 illustrates the movements of cells before and after these overlap to form a *clump*. Red lines indicate the movement of each cell before they interact and overlap to form a *clump*, and the green lines indicate the movement after. In some cases, the cells barely move from the point where the interaction started (*clump* 2001 in Figure 6), whilst in others, the cells seem to *cross over each other* and continue their paths far from where the interaction began (*clump* 22001 in Figure 6). In this work, we consider that the change of orientation is reflected by the directions before and after the *clump* is formed and will not consider the movement within the *clump* itself.

**Figure 7:**
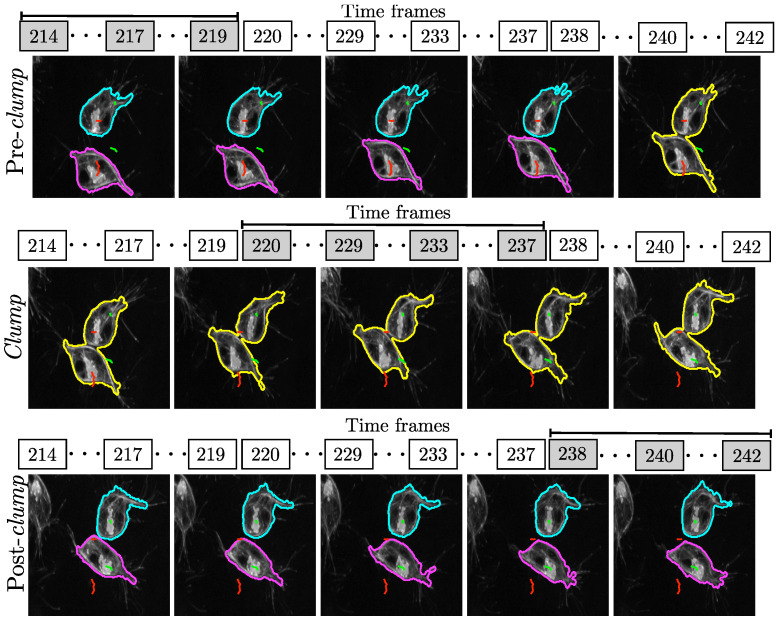
Representation of the migration of two cells as they form a *clump*. The perimeters of the individual cells are highlighted by cyan and magenta lines, whilst the perimeter of the *clump* is highlighted with a yellow line. Red lines indicate the movement of the individual cells before the clump is created, and a green line indicates the positions of cells after they separate. To show the duration of the *clump*, the number of time frames is shown above the images. In this case, the cells overlap and form the *clump* for 18 frames, which is equivalent to 180 s.

In addition, to illustrate these movements over a sequence of time frames, Figure 7 follows a pair of cells during several frames to illustrate the formation of the clump and the return to individual cells. The number of frames in which the cells appear in a *clump* is relevant to the study of the movement as it acts as a proxy for the time cells were in actual contact (ten seconds per frame).

**Figure 8:**
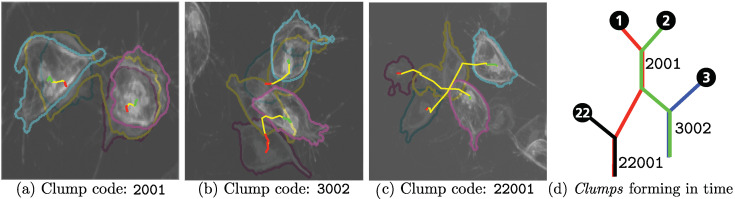
Frames in different interactions overlapped to appreciate cell movement and *clump* formation. (**a**–**c**) Three frames are superimposed: the first, middle, and final frames in each experiment are shown, with corresponding segmentations and tracks. The full track in each experiment is presented, with changes of colour representing different moments: before (red), during (yellow), and after (green) the *clump*. (**d**) is a representation of the same cells forming different *clumps* at different time points.

The cells in Figure 7 are shown in the clump for 18 frames (3 min). It is worth noting that a single clump could provide more than one experiment in different time spans, as the two interacting tracks could interact with each other back and forth. An illustration of one interaction is shown in Figure 8.

### 3.1. Analysis of Movements and Interactions

**Figure 9:**
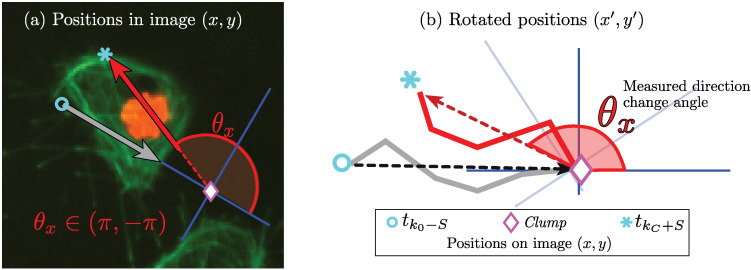
Illustration of direction change ($\theta_{x}$) measurement. Three markers represent different positions of a given track. The markers are as follows: (∘) represents *S* frames before contact; (⋄) represents the starting instant of the clump; and (*) represents the position where the experiment is finalised. Notice the translation and rotation into the new frame of reference $\left(x^{\prime },y^{\prime }\right)$.

The events of interest in this paper consist of analysing the cell-cell contact events of two cells, and these will be called *interactions*. The change of direction $\theta_{x}\in \left(-\pi ,\pi \right)$ is calculated by taking the positions of the tracks $T_{r}$ and $T_{q}$ up to *S* frames prior to the first contact at time frame $t_{k_{0}}$, as well as the positions up to *S* frames after the last time frame of contact $t_{k_{c}}$. The time in clump $TC=t_{k_{c}}-t_{k_{0}}$ refers to the number of frames in which the two tracks interact in a given instance of the clump, and it is not taken into consideration for the calculation of angle $\theta_{x}$. A diagram of the calculation of $\theta_{x}$ is provided in Figure 9a. For visualisation purposes, the reference axes are translated and rotated from the positions on the image x=(x,y) to a new frame of reference $\left(x^{\prime },y^{\prime }\right)$ so that the path of the cell before the interaction is always travelling from left to right and aligned with the horizontal axis. In this way, it is possible to compare the changes in direction of any pair of cells.

### 3.2. Selection of Interactions

All available datasets were segmented and tracked in both fluorescent channels. A careful analysis of the tracks was performed to determine the cases where cells overlapped in the green channel to form a *clump*. In addition, to consider an interaction, the following criteria were applied: (i) only two cells were present in the current *clump*; (ii) full interaction, where at least one of the cells would enter and exit the *clump*. The interest of this work was to determine an immediate reaction after leaving the *clump*; thus, the range of values of *S* that was considered was between three and five, which corresponded to 30 to 50 s. Longer values of *S* could observe more long-term variation and could be the subject of a different study.

For those cases where the conditions were met, the following parameters were measured: variation of direction angle $\theta_{x}$ and time in clump TC.

## 4. Results

After the processes of segmentation, tracking, and selection of suitable interactions, twenty four control and thirty nine mutant interactions were selected for analysis. These were present in four of the five control datasets and eight of the eleven mutant datasets.

Table 1 shows the number of interactions per dataset selected. It is important to observe that any interactions of three or more cells were not considered, and this could impact into the number of interactions per dataset. Whilst the differences could correspond to a biological difference between the datasets, that analysis is not within that scope of the present work.

**Figure 10:**
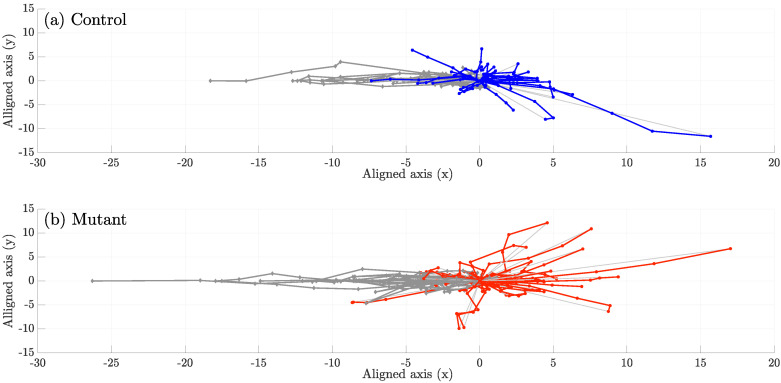
Comparison of aligned tracks for (**a**) control and (**b**) mutant interactions. Each line represents the trajectory of one cell, and the marker (·) represents the position at a certain time frame. Each line can be read from the utmost left point and continuing, initially towards the right, along the line to the next time frame marker. The grey lines correspond to the cells before entering *clump*, where the origin (0,0) corresponds to the clump formation. Red and blue lines correspond to five time frames of each cell after exiting the clump.

The number of interactions per dataset averaged 6±5.41 for controls and 4.87±3.31 for mutants. The resulting tracks representing changes of direction are shown in Figure 10 for (a) control and (b) mutant. Differences can be observed in the displacement of the cells towards and from the centre or origin of the new reference frame in Figure 9, in the horizontal direction $x^{\prime }$.

**Figure 11:**
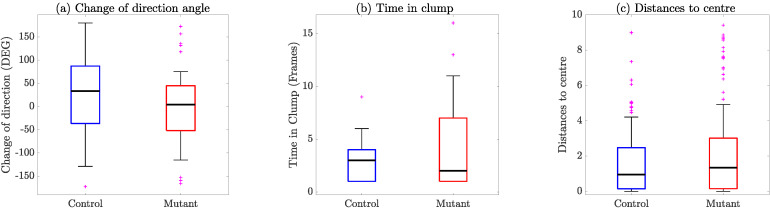
Comparison of relevant variables between control (blue) and mutant (red) interactions. (**a**) Change of direction angle, $\theta_{x}$, coming from Figure 10. (**b**) Time in clump TC in frames. Finally, (**c**) shows the distances to the centre or origin of the new frame of reference $\left(x^{\prime },y^{\prime }\right)$ (i.e., the length of the tracks after they leave the clump).

The first hypothesis to be tested was to see if cells tended to move more towards one side (e.g., left) or another. For this, we compared the change of direction when $-180^{\circ }<\theta_{x}<180^{\circ }$ (Figure 11a). Whilst it appeared that the angle $\theta_{x}$ for mutant interactions was distributed towards the lower angles, or a smaller change of direction after the contact, there was no statistical significance between these cases. Similarly, there were no statistical differences for the time in clump TC (Figure 11b) and distances from the origin of the $x^{\prime },y^{\prime }$ coordinate plane (Figure 11c).

**Figure 12:**
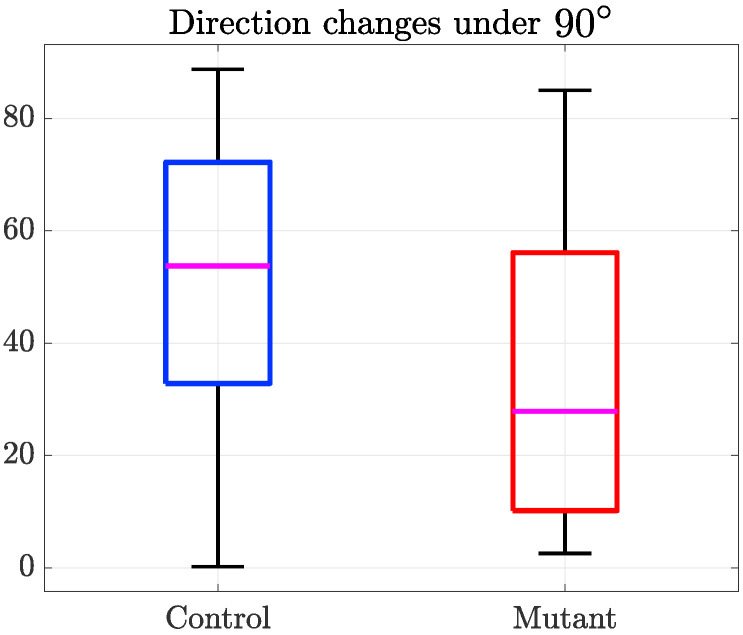
Change of direction differences between control (blue) and mutant (red) interactions for those tracks whose absolute value of the angle is in the range $0^{\circ }<\theta_{x}<90^{\circ }$. These two populations present a statistical significant difference (p=0.03).

The next hypothesis to be tested was to compare the change of direction in absolute terms, i.e., not considering left and right, only the angle, and only for the range that would constitute a change of the direction more than a repulsion. In other words, we only considered those tracks where $-90^{\circ }<\theta_{x}<90^{\circ }$, and by taking the absolute value of the angle, we discarded the sign, which resulted in the following range $\left(abs\left(\theta_{x}\right)<90\right)$. Figure 12 shows the distribution of these values for each population. For this case, a *t*-test indicated statistical significance (p=0.03<0.05) between the controls and mutants, suggesting that controls varied their direction with higher angles than mutants.

## 5. Discussion

This work presented a comparison of the movement that follows a contact between two cells. Migrating control and mutant Drosophila embryonic macrophages were imaged by fluorescence microscopy and their interactions quantified.

The observation of such datasets indicated that the number of interactions found per dataset was not always consistent. In many cases, problems with the segmentation of the fine microtubule arm-like structures described in [19] could be lost due to the post-processing stages of the segmentation. In particular, with these datasets, the focus would vary extensively (Figure 2), complicating part of the analysis. Whilst the number of interactions that were selected from the datasets was small, there was an indication that there could be differences between the mutant and the control cells in the sense that the control cells showed a greater change of direction after interaction than the mutants. However, to obtain this result, it was necessary to select only interactions under specific conditions, as seen in Figure 12. The results were encouraging and suggested that studies with larger samples should be performed in order to confirm this observation for a relatively small population. The tools developed in this paper could be used for these larger studies.

## 6. Conclusions

This work presented a description of the software macrosight, as a tool to analyse the movement of cells, in particular with respect to the change of direction after contact between cells. The software macrosight was demonstrated with the analysis between two different cell populations: control and *Shot3* macrophages. While encouraging results were found, the differences between cell populations were only statistically significant in very specific conditions. Future work will concentrate on increasing the number of datasets, which will in turn increase the number of interactions. Additionally, a larger number of variables collected from the tracking should be explored, and the segmentation could be enhanced with a step detecting discrete alignment of microtubules, therefore increasing the accuracy of interactions detected.

## Figures and Tables

**Table 1:** Number of suitable interactions per dataset Each dataset corresponded to one *Drosophila* embryo. Initially, five control and 11 mutant datasets were analysed. Of these, one control (04) and three mutant datasets (01, 02, 09) did not provide any suitable interactions, mainly due to *clumps* that were formed by more than two cells. The different number of interactions per dataset should be noticed, which was due to the variability of the cell interactions.

Dataset ID	*n* Interactions	Dataset ID	*n* Interactions	Dataset ID	*n* Interactions
CONTROL01	14	MUTANT03	10	MUTANT07	3
CONTROL02	4	MUTANT04	2	MUTANT08	2
CONTROL04	4	MUTANT05	2	MUTANT10	4
CONTROL05	2	MUTANT06	9	MUTANT11	7
TOTAL	24	TOTAL	39		

## Abbreviations

The following abbreviations are used in this manuscript:

ΔDirection	Change of direction
GFP	Green fluorescent probe
MIUA	Medical Image Understanding and Analysis
$T_{i}$	Track acquired with identifier *i*
TC	Time in clump
